# Hwangryunhaedok-Tang Exerts Neuropreventive Effect on Memory Impairment by Reducing Cholinergic System Dysfunction and Inflammatory Response in a Vascular Dementia Rat Model

**DOI:** 10.3390/molecules24020343

**Published:** 2019-01-18

**Authors:** Eunjin Sohn, Yu Jin Kim, Hye-Sun Lim, Bu-Yeo Kim, Soo-Jin Jeong

**Affiliations:** 1 Clinical Medicine Division, Korea Institute of Oriental Medicine, Daejeon 34054, Korea; ssen4022@kiom.re.kr (E.S.); jinjin0228@kiom.re.kr (Y.J.K.); qp1015@kiom.re.kr (H.-S.L.); buykim@kiom.re.kr (B.-Y.K.); 2 College of Pharmacy, Chungnam National University, Daejeon 34134, Korea

**Keywords:** Hwangryunhaedok-tang, vascular dementia, bilateral common carotid artery occlusion, acetylcholinesterase, anti-neuroinflammatory effect

## Abstract

Hwangryunhaedok-tang (HRT) is a traditional oriental herbal formula used in Asian countries for treating inflammatory diseases and controlling fever. Our present study aimed to determine whether HRT has therapeutic effects for patients with vascular dementia (VaD) using a bilateral common carotid artery occlusion (BCCAO) rat model and assessing spatial memory impairment and activation of neuroinflammation. BCCAO was performed in male Sprague Dawley rats to induce VaD, and oral HRT was administered daily for 30 d. Our data showed that HRT ameliorated BCCAO-induced memory and cognitive impairment in behavioral tests. In addition, HRT reversed cholinergic dysfunction and neuronal damage in the hippocampus of BCCAO rats. Furthermore, HRT attenuated microglial activation and reduced the phosphorylation of p38 mitogen-activated protein kinase and c-Jun N-terminal kinase (JNK) induced by BCCAO. Simultaneous high-performance liquid chromatography analysis of HRT using index compounds from the herbal composition revealed that both HRT ethanol extract and commercial HRT granules primarily comprise geniposide, baicalin, and berberine. Our study showed that HRT administration resulted in the prevention of neuronal injury induced by BCCAO through improvement of cholinergic dysfunction and inhibition of neuroinflammatory responses, suggesting that HRT may have potential as a treatment for VaD.

## 1. Introduction

Hwangryunhaedok-tang (HRT) in Korea, called Hwanglianjiedu-tang in China and Orengedokuto in Japan, is a traditional herbal formula comprising four medicinal herbs in a 1:1:1:1 ratio: Phellodendri Cortex, Scutellariae Radix, Coptidis Rhizoma, and Gardeniae Fructus. HRT is used clinically for reducing fever and treating various inflammatory diseases [1]. In addition, recent studies demonstrated that HRT exerts biological activities on chronic obstructive pulmonary disease [2], cerebral ischemia [3], arthritis [4], gastric injury [5], and gastrointestinal motility function [6]. 

Dementia is described as a set of symptoms including memory loss and difficulties with thinking, problem-solving, or language [7]. Vascular dementia (VaD) is the second most common dementia after Alzheimer’s disease (AD) and accounts for about 15% of all cases [8]. Chronic cerebral hypoperfusion is known to be a major cause of VaD, leading to cognitive impairment. However, the pathogenesis of VaD is still not fully understood, and there are no approved medications for VaD [9]. Interestingly, several groups reported a cholinergic deficit in the cerebrospinal fluid and brain that may be associated with the pathogenesis of VaD [10,11,12]. Thus, cholinergic drugs such as acetylcholinesterase (AChE) inhibitors are suggested as promising therapies for VaD [13]. 

Several clinical and nonclinical investigations suggested that HRT has therapeutic efficacy in AD treatment [14,15,16,17]. However, the effects of HRT on VaD are yet to be reported. In this study, we investigated the therapeutic effects of HRT on VaD using a bilateral common carotid artery occlusion (BCCAO) rat model by assessing spatial memory impairment and activation of neuroinflammation. 

## 2. Results

### 2.1. Inhibitory Effects of the Ethanol Extract of HRT and Commercial HRT Granules on AChE Activity

**Figure 1:**
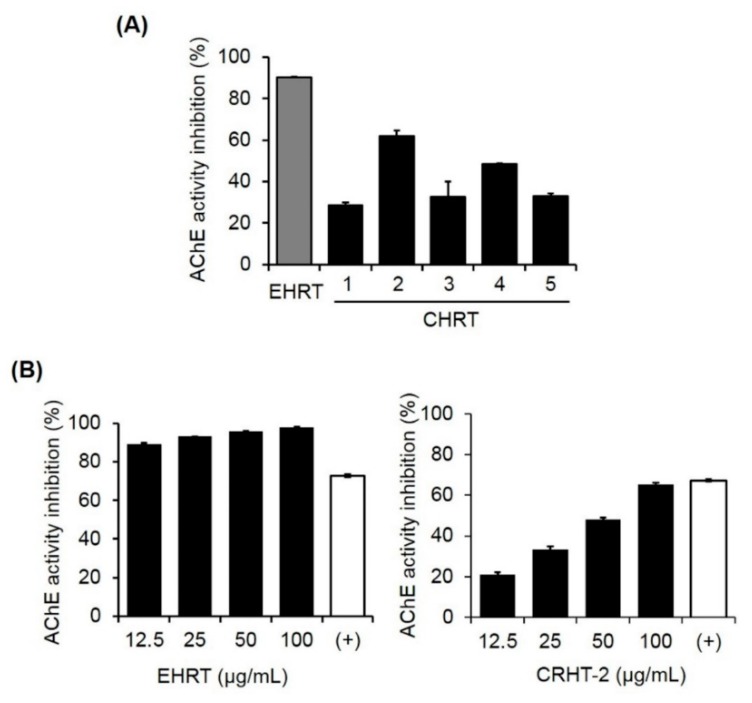
Inhibitory effects of the ethanol extract of Hwangryunhaedok-tang (EHRT) and five commercial HRT granules (CHRTs 1–5) on acetylcholinesterase (AChE) activity. The AChE activity assay was performed using a modified Ellman’s colorimetric method. The enzymatic reaction was performed by incubating the mixture of AChE solution with (**A**) EHRT or CHRTs 1–5 (100 μg/mL); (**B**) EHRT (12.5, 25, 50, or 100 μg/mL) (left panel); and CHRT-2 (12.5, 25, 50, or 100 μg/mL) (right panel) for 1 h at room temperature. Absorbance was measured at 412 nm using an Epoch microplate spectrophotometer (Bio-Tek Instruments, Winooski, VT, USA). Berberine (500 nM) was used as a positive control (+). Each value is presented as the mean ± standard error of the mean (SEM) (*n* = 3).

AChE enzymatic activity was assessed with an assay for comparison of the biological activity of ethanol extract of HRT (EHRT) and five commercial HRT granules (CHRT-1 through CHRT-5) on AChE activation. As shown in Figure 1A, EHRT dramatically inhibited AChE activity by 90.13% compared with the control. Among the five CHRTs, CHRT-2 had the most robust inhibitory effect on AChE activation (62.06% inhibition). CHRTs 1, 3, 4, and 5 produced <50% inhibition. To further confirm the inhibitory effects of EHRT and CHRT-2 on AChE activity, the assay was again conducted at lower concentrations (12.5 to 100 µg/mL). As shown in Figure 1B, considerable AChE inhibitory activity was shown even at 12.5 µg/mL EHRT (left panel). CHRT-2 also decreased AChE activity in a dose-dependent manner (right panel). 

### 2.2. Effect of HRT on Cognitive Impairment in the BCCAO-Induced VaD Rat Model

**Figure 2:**
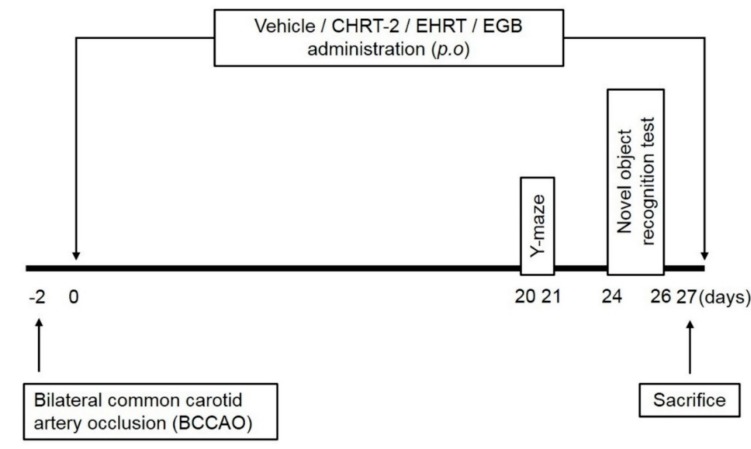
Schematic description of the experimental design. Sprague Dawley rats were randomly assigned to six experimental groups (*n* = 8 per group). The experimental group comprised (1) the sham normal group, (2) the bilateral common carotid artery occlusion (BCCAO) group, (3) the BCCAO group treated with CHRT-2 at 200 mg/kg, (4) the BCCAO group treated with CHRT-2 at 400 mg/kg, (5) the BCCAO group treated with EHRT at 400 mg/kg, and (6) the BCCAO group treated with an extract of *Ginkgo biloba* (EGB) at 100 mg/kg. Oral administration of drugs was started 2 d after BCCAO induction and continued for 30 days. Y-maze and novel object recognition tests were conducted 24 h after last administration at 23 and 27 days, respectively.

**Figure 3:**
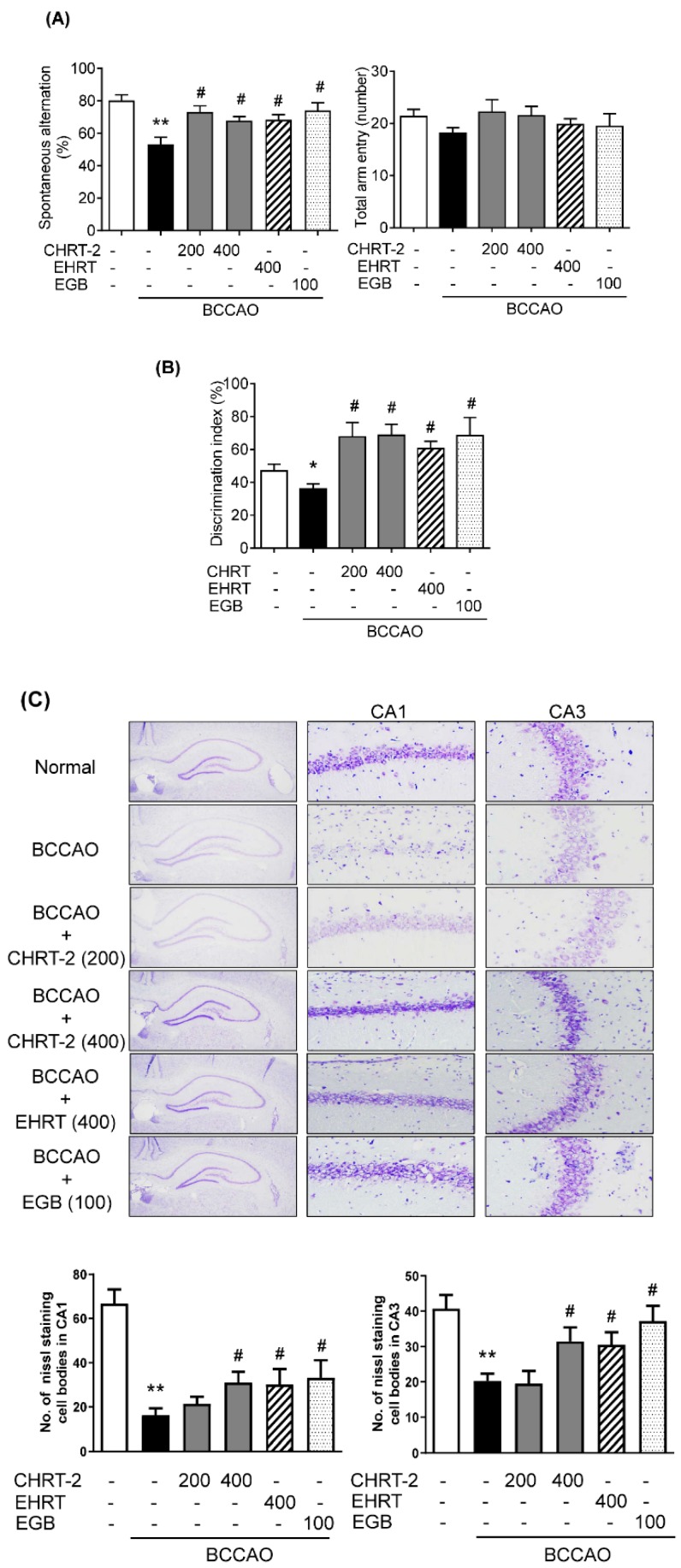
Effects of HRT on memory and cognitive impairment in BCCAO-induced vascular dementia (VaD) rats. (**A**) For the Y-maze test, the spontaneous alternation behavior (left) and the number of total arm entries (right) were monitored during a 10-min session. (**B**) For the novel object recognition test, the discrimination ratio was calculated as the ratio of time spent exploring the novel object or the object moved to a novel location to the total time spent exploring. Data are presented as means ± SEM; ** *p* < 0.01 vs. normal group, # *p* < 0.01 vs. BCCAO group. (**C**) Sections of the hippocampal Cornu Ammonis 1 and 3 (CA1 and CA3) regions were prepared and used for Nissl staining using cresyl violet solution. The graph shows the number (No.) of Nissl staining cells in CA1 and CA3. Representative photomicrographs are at magnifications of 400×.

Figure 2 presents a schematic histogram of the animal experiments. To determine the effect of HRT on cognitive deficits, behavioral tests were performed using BCCAO-induced VaD rats. In the Y-maze test, BCCAO induction significantly decreased spontaneous alternation behavior compared to that shown in the normal group. In contrast, treatment of EHRT or CHRT-2 significantly reversed the decreased levels of spontaneous alternation caused by BCCAO (Figure 3A, left panel). The total number of arm entries was relatively similar across all experimental groups (Figure 3A, right panel). In the novel object recognition task, the normal group successfully discriminated between the familiar and novel objects, while the BCCAO group had a somewhat higher failure rate (*p* > 0.05). In contrast, administration of EHRT or CHRT-2 to BCCAO rats significantly increased the percentage of discrimination for the novel object compared with that of the BCCAO group (Figure 3B). There was no significant different between HRT and CHRT-2 in terms of cognition enhancement. An extract of *Ginkgo biloba* (EGB) was used as a positive control [18].

### 2.3. Protective Effect of HRT against Neuronal Cell Loss in the BCCAO-Induced VaD Rat Model

The neuroprotective effect of HRT in a VaD rat model was identified by Nissl staining. As shown in Figure 3C, considerable cell loss was observed in the Cornu Ammonis (CA)1 and CA3 regions of the hippocampus of the BCCAO group. EHRT, CHRT-2, or EGB administration resulted in dramatic increases in the number of surviving cells in the hippocampal subfields compared with numbers observed in the BCCAO group.

### 2.4. Effect of HRT on BCCAO-Induced Modification of Acetylcholine (ACh) Level and AChE Activity in Rats

**Figure 4:**
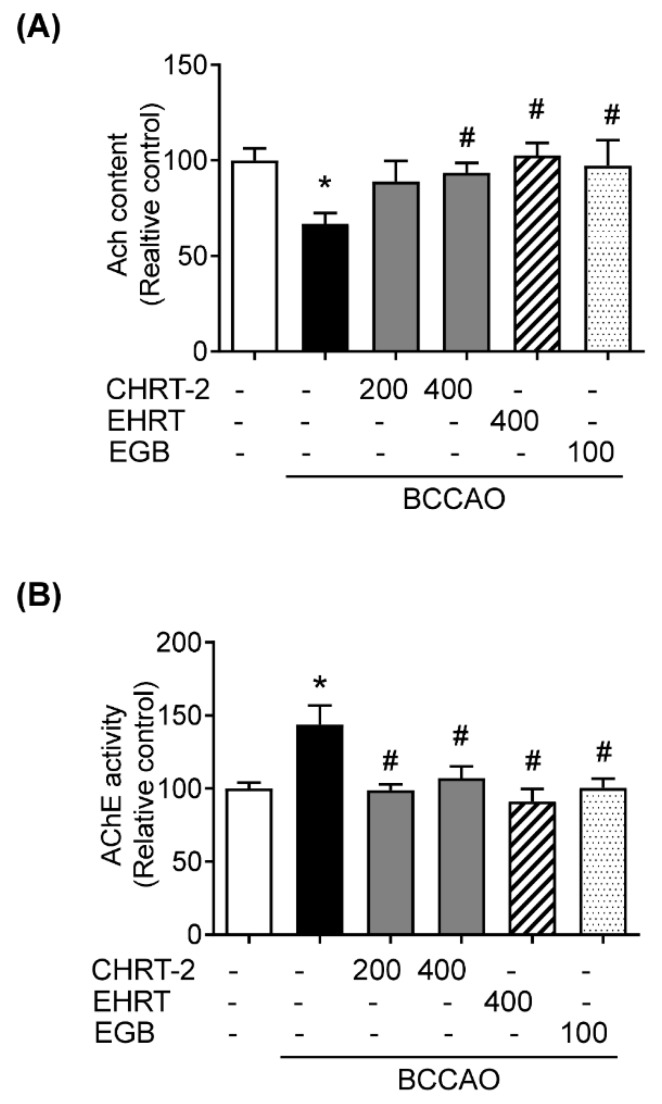
Effect of HRT on cholinergic system dysfunction in BCCAO-induced VaD rats. Acetylcholine (ACh) content (**A**) and AChE activity (**B**) were measured using an ACh and AChE activity assay kit (US Biomax Inc., Derwood, MD, USA). Data are presented as means ± SEM (*n* = 8); ** *p* < 0.01 vs. normal group, # *p* < 0.01 vs, BCCAO group

Cholinergic deficiency is involved in the pathogenesis of VaD [19]. Thus, the levels of ACh and AChE activity in the hippocampus were measured to determine whether HRT affects cholinergic deficiency in BCCAO-induced VaD mice. The results revealed that the ACh level in the BCCAO group was markedly reduced compared to that in the normal group. Treatment of EHRT, CHRT-2, or EGB to BCCAO mice induced the recovery of ACh levels (Figure 4A). Treatment with EHRT or CHRT-2 significantly and consistently blocked the BCCAO-induced activation of AChE (Figure 4B).

### 2.5. Effect of HRT on Glial Cell Activation in the BCCAO-Induced VaD Rat Model

**Figure 5:**
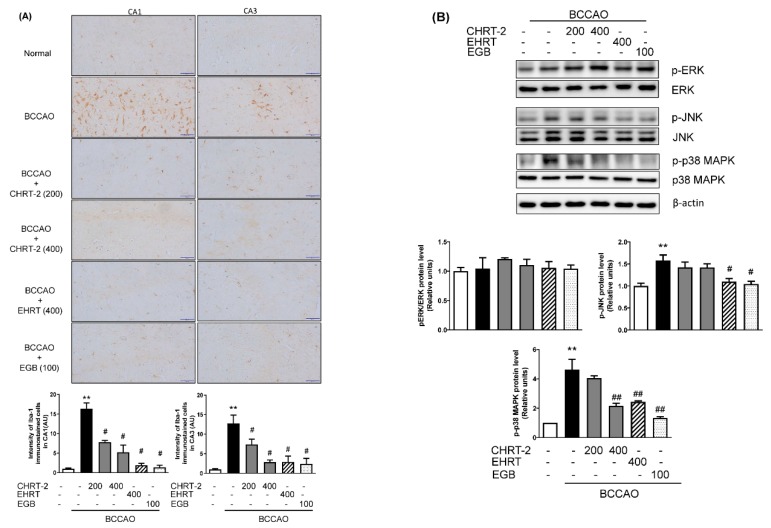
Effect of HRT on inflammatory responses in the BCCAO-induced VaD rats. (**A**) Immunohistological analysis was carried out to examine the expression of ionized calcium binding adaptor molecule 1 (Iba-1), a microglial marker, in the hippocampus of BCCAO rats. Representative photomicrographs are shown at magnifications of 400×. The immunohistochemical signal intensity was calculated in a brain tissue section from each rat using Image J software. The staining intensity results are reported in arbitrary units (AU). (**B**) Hippocampal tissues were lysed and subjected to Western blotting for detecting phosphorylated mitogen-activated protein kinases (MAPKs) including extracellular signal-regulated kinase (ERK), c-Jun N-terminal kinase (JNK), and p38 MAPK. Expression levels were normalized to the expression of actin. Data are presented as means ± SEM (*n* = 8); ** *p* < 0.01 vs. normal group, # *p* < 0.05 and ## *p* < 0.01 vs. BCCAO group.

In addition to cholinergic deficiency, inflammation in response to ischemic injury occurs in VaD [20]. To quantify microglia, a key cellular mediator of neuroinflammatory response [21], immunohistochemical analysis was performed using anti-ionized calcium binding adaptor molecule 1 (Iba-1) antibody, an activation marker for microglia. In the normal group, microglia in the hippocampal tissues revealed a scattered of ramified form, indicating an inactivated state. In the BCCAO group, microglia in the CA1 and CA3 regions were hypertrophied and condensed, indicating an active state. Treatment with HRT, CHRT-2, or EGB markedly inhibited the increase in activated microglia compared with the BCCAO group (Figure 5A).

### 2.6. Effect of HRT on Mitogen-Activated Protein Kinase (MAPK) Signaling in the BCCAO-Induced VaD Rat Model

MAPK signaling plays a crucial role in neuroinflammatory regulation, and its upregulation was observed in the BCCAO rat brain tissue [4,22]. To examine the effects of HRT on the activation of MAPK signaling, the phosphorylated levels of extracellular signal-regulated kinase (ERK), p38 MAPK, and c-Jun N-terminal kinase (JNK) of the MAPK family were determined by Western blotting. As shown in Figure 5B, the phosphorylated JNK and p38 MAPK relative protein levels—but not that of ERK—were increased in the BCCAO group compared to normal group. In the HRT groups, EHRT administration had no significant effect on phosphorylated JNK relative protein level, but reduced the phosphorylated p38 MAPK protein level. CHRT-2 treatment revealed suppressive effects on the JNK and p38 MAPK phosphorylation level compared with the BCCAO group. EGB administration reduced the BCCAO-mediated phosphorylation of p38 JNK and p38 MAPK protein.

### 2.7. High-Performance Liquid Chromatography (HPLC) Determination of the Seven Components in HRT

**Figure 6:**
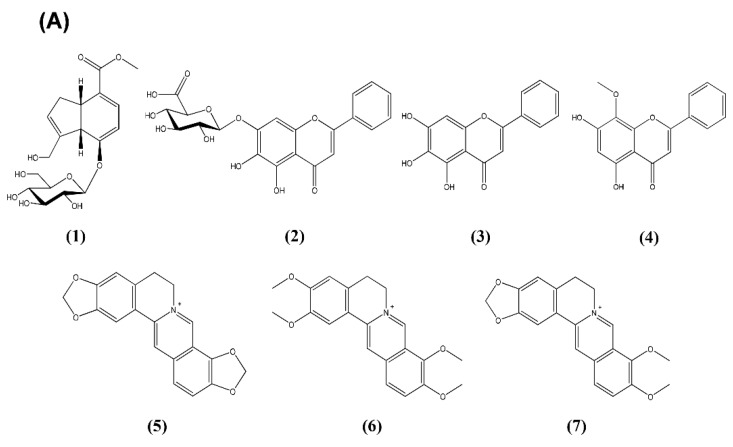

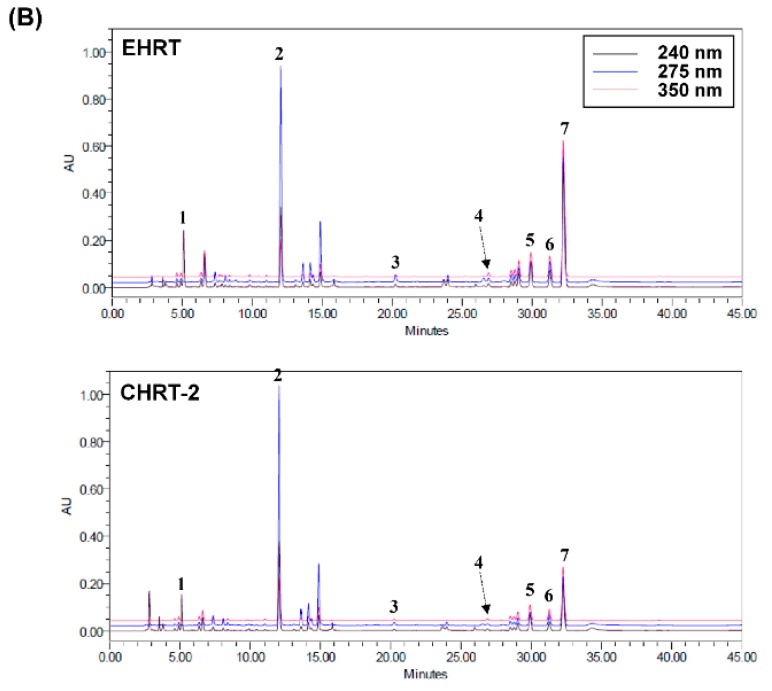
Chemical structures of the seven standard compounds (**A**) and HPLC chromatograms of HRT formulations (**B**). Simultaneous quantification of EHRT (2 mg/mL) and CHRT-2 (10 mg/mL) was conducted at 240, 275, and 350 nm. 1: geniposide, 2: baicalin, 3: baicalein, 4: wogonin, 5: coptisine, 6: palmatine, and 7: berberine.

The optimized method for HPLC analysis was used for simultaneous determination of the seven standard components (Figure 6A) in HRT. The best chromatographic separation was obtained using mobile phases consisting of 10% (*v*/*v*) acetonitrile in 0.2% SDS with 0.02% phosphoric acid (A) and acetonitrile (B). The ultraviolet (UV) wavelengths to detect components were 240 nm for geniposide; 275 nm for baicalin, baicalein, and wogonin; and 350 nm for coptisine, palmatine, and berberine. The seven components were resolved within 33 min under the established HPLC methods. The retention times for geniposide, baicalin, baicalein, wogonin, coptisine, palmatine, and berberine were 5.11, 12.03, 20.24, 26.52, 29.9, 31.26, and 32.22 min, respectively. HPLC chromatograms for EHRT and CHRT-2 are presented in Figure 6B. The levels of geniposide, baicalin, baicalein, wogonin, coptisine, palmatine, and berberine in EHRT were 50.19, 99.92, 3.16, 1.65, 15.39, 12.06, and 93.74 mg/g, respectively (upper panel). HPLC analytical patterns for CHRT-2 were similar to those for EHRT (lower panel).

## 3. Discussion

VaD is the second most common type of dementia caused by blocking blood flow to the brain tissue (www.healthdirect.gov.au/vascular-dementia). The present study demonstrates that the traditional herbal formula HRT alleviates cognitive impairment and hippocampal damage via activation of the cholinergic system and stimulation of phosphorylated JNK and p38 MAPK signaling in BCCAO rats. BCCAO, the most popular VaD mimic animal model, is achieved by global chronic cerebral hypoperfusion via permanent occlusion of both common carotid arteries [23,24]. The BCCAO model exhibits neuronal damage in the brain, inflammatory reaction, and blood–brain barrier disruption [24]. In various animal behavioral tests, the BCCAO model exhibits memory or cognitive impairment, a key indicator of dementia [25,26]. Our histochemical analysis consistently demonstrated that rats in the BCCAO group exhibited substantial neuronal damage in the CA1 and CA3 regions of hippocampus. Oral administration of HRT prevented BCCAO-induced neuronal damage at 400 mg/kg EHRT or CHRT-2 compared with the BCCAO group. Moreover, BCCAO induced a significant decline in spontaneous alternation in the Y-maze test and in the novel object recognition test as compared with the normal group. In contrast, HRT administration mitigated poor performance induced by BCCAO. There was no significant difference between EHRT and CHRT-2 regarding effects on memory enhancement.

Although the precise mechanisms involved in VaD remain unclear, cholinergic deficits in the brain constitute a critical characteristic of VaD patients [27,28]. Central cholinergic dysfunction triggers memory and cognitive difficulties in VaD patients [19]. Therefore, cholinergic therapies are suggested to ameliorate VaD. In our experiments, BCCAO-induced rats had significantly lower ACh levels and had higher AChE activity than did normal rats. Treatment with EHRT or CHRT-2 significantly restored ACh levels and AChE activity compared with the BCCAO group. We also confirmed the inhibitory effect of HRT on AChE activation using an in vitro enzymatic activity assay. EHRT and CHRT-2 both increased AChE activity in a dose-dependent manner. These data indicate the potential of HRT as a cholinergic agent for VaD treatment.

Neuronal inflammation plays a key role in the pathogenesis of VaD [20,29]. Many studies reported on inflammatory responses such as the activation of microglia, which are major inflammatory cells in the brain [21,30], and inflammation-related signaling in BCCAO rats [22,31]. In line with previous reports, our findings demonstrated that HRT administration decreased the number of positive cells stained with anti-Iba-1, a marker of microglia [32], induced by chronic BCCAO in the hippocampal CA1 and CA3 regions, indicating the anti-neuroinflammatory activity of HRT. MAPK signaling is implicated in the activation of microglia [22,33] and is also closely associated with dysfunction of the cholinergic system [19]. The present study showed that administration of EHRT decreased the levels of phosphorylated JNK and p38 MAPK in the hippocampus. However, CHRT-2 had an inhibitory effect on the phosphorylation of p38 MAPK, but not on JNK. The differing results between EHRT and CHRT-2 may be due to carriers used in the manufacture of CHRT-2. Both EHRT and CHRT-2 commonly mediated changes in p38 MAPK phosphorylation. Anti-neuroinflammatory effects of HRT may be related to the traditional usage of HRT for inflammatory diseases.

HRT comprises four medicinal herbs: Phellodendri Cortex, Scutellariae Radix, Coptidis Rhizoma, and Gardeniae Fructus. The main ingredients of HRT are benzylisoquinoline alkaloids (e.g., coptisine, palmatine, and berberine) from Coptidis Rhizoma and Phellodendri Cortex [34,35], flavonoids (e.g., baicalin, baicalein, wogonin, and wogonoside) from Scutellariae Radix [36], and iridoid glycoside (e.g., geniposide and geniposidic acid) from Gardeniae Fructus [37]. We conducted simultaneous determinations of the seven components (geniposide, baicalin, baicalein, wogonin, coptisine, palmatine, and berberine) in EHRT using an HPLC photodiode array (PDA) method. Consequently, baicalin, berberine, and geniposide were abundant in EHRT and in CHRT-2. Previous reports suggested that these compounds have beneficial effects on VaD patients. Zhang et al. reported the effects of baicalin and berberine from HRT on ischemic stroke [38]. Li et al. reported the effect of genoposide on VaD in a chronic cerebral hypoperfusion rat model [39]. In general, a sudden disruption of the blood supply to distinct brain regions leads to stroke, while a moderate but persistent reduction in regional cerebral blood flow compromises memory processes and contributes to the development and progression of dementia. The association of decreased cerebral blood flow (CBF) with VaD was firmly established [40,41,42].

Many studies reported the possibility of using herbal medicines as VaD medications [43,44,45], and herbal medicines are used in traditional oriental medicine to treat stroke or VaD in modern medicine. Further studies will be necessary to identify a bioactive compound of HRT that controls VaD pathogenesis.

In conclusion, we demonstrated that the herbal formula HRT alleviates memory impairment and cholinergic dysfunction induced by BCCAO. HRT exerts anti-inflammatory effects via inhibition of p38 MAPK phosphorylation in the hippocampus of BCCAO rats. These findings suggest that HRT ameliorates neurological dysfunction and memory impairment by improving cholinergic system function and preventing inflammation in a VaD-like animal model. Our results suggest that HRT has potential as a therapeutic agent for controlling VaD.

## 4. Materials and Methods

### 4.1. Herbal Materials

HRT comprised four herbs: Phellodendri Cortex, Scutellariae Radix, Coptidis Rhizoma, and Gardeniae Fructus. These herbal materials were obtained at the herbal commercial market (Kwangmyungdang, Ulsan, South Korea). A voucher specimen (SCD-B-022) was deposited at the Clinical Medicine Division (Korea Institute of Oriental Medicine, Daejeon, Korea).

### 4.2. Preparation of HRT Samples

The four herbs—Phellodendri Cortex (10 g), Scutellariae Radix (10 g), Coptidis Rhizoma (10 g), and Gardeniae Fructus (10 g)—were mixed and extracted with 240 mL of 70% aqueous ethanol (2 h, twice) by refluxing. The extracted solution was filtered and concentrated using a rotary evaporator system under vacuum. The powdered EHRT (11.99 g) was obtained with a yield of 29.98%. CHRT-1 through CHRT-5 were obtained from Kyoungbang Pharm. Co., Ltd. (Incheon, Korea), Tsumura & Co. (Chou-ku, Tokyo, Japan), Hanpoong Pharm & Foods Co., Ltd. (Jeonju, Jeonbuk, Korea), Han Kook Shin Yak Pharmaceutical Co., Ltd. (Nonsan, Chungnam, Korea), and I-World Pharm Corp. (Incheon, Korea), respectively, and were dissolved in phosphate-buffered saline (PBS) for both in vivo and in vitro experiments. EHRT and CHRT-2 were weighed and dissolved in 10% aqueous dimethyl sulfoxide (DMSO) at 2 and 10 mg/mL, respectively, and then passed through a syringe filter (0.45 μm) to use as samples for HPLC analysis.

*Ginkgo biloba* extract (EGB) was obtained from Vitacost Supplement Co. (Las Vegas, NV, USA). Each pill contained 28.8 mg (24%) of ginkgo flavonglycosides and 7.2 mg (6%) of terpene lactones. EGB was used as a positive control and, based on previous study [46], we assumed that the minimum effective dose of EGB was 100 mg/kg body weight in the BCCAO animal model.

### 4.3. In Vitro Assay for AChE Inhibitory Activity

To investigate the in vitro AChE inhibition activity, the manufacturer’s protocol for the Acetylcholinesterase Assay Kit (Abcam, Cambridge, UK) based on a modified Ellman’s colorimetric method [47] was used. The stock solutions of EHRT and five CHRTs in DMSO (100 mg/mL) were diluted with 0.1 M sodium phosphate buffer (pH 8.0) to 100 μg/mL to use as sample solutions. These sample solutions was then serially diluted with the same buffer to final concentrations of 100, 50, 25, and 12.5 μg/mL. AChE in 0.1% bovine serum albumin/H_2_O (25 U/mL) was dissolved in assay buffer (0.1 M sodium phosphate buffer, pH 7.3) to 35.2 mU/mL final concentration. To prepare the reaction mixture, the substrates 5,5′-dithiobis-(2-nitrobenzoic acid) and acetylthiocholine iodide were dissolved in assay buffer and H_2_O, respectively, to 10 mM, and then mixed in assay buffer to 0.5 mM final concentration. To assess the enzymatic reaction, the sample solution (50 µL) and the reaction mixture (50 µL) were mixed in 96-well plates, and pre-incubated (10 min) at room temperature. The AChE solution (10µL) was treated to each well to start the enzymatic reaction, which was conducted for 60 min at 10-min intervals and measured using an Epoch microplate spectrophotometer (Bio-Tek Instruments, Winooski, VT, USA) at 412 nm. All experiments were conducted in triplicate, and berberine was used as a positive control [48]. The percentage inhibitory activity of AChE was calculated using the following equation:

(1) $$\mathrm{AChE}\;\mathrm{activity}\;\mathrm{inhibition}\;(\%)=1-\frac{S-S’}{C-C’}\times \;100,$$

where S is the sample volume (reaction mixture, sample solution, and enzyme), S’ is the sample volume without enzyme (reaction mixture, sample solution, without enzyme), C is the control volume (0.1 M sodium phosphate buffer (pH 8.0), reaction mixture, and enzyme, and C’ is the control volume without enzyme (0.1 M sodium phosphate buffer (pH 8.0), reaction mixture, without enzyme).

### 4.4. Animal Preparation and Surgical Procedures

Male Sprague Dawley rats (250–300 g, OrientBio Inc., Seoungnam, Korea) were used for this study. The rats were housed in a controlled environment at the Korea Institute of Oriental Medicine (22 ± 1 °C; humidity 55 ± 10%; 12-h dark/light cycle) and were allowed water and food freely throughout the experiment. The experiments were performed in accordance with the Guide for the Animal Care and Use of Laboratory Animals as adopted by the National Institutes of Health guidelines. The protocol was approved by the Committee of the Korea Institute of Oriental Medicine (Approval No. 18-001). BCCAO procedures were performed as described previously [49]. In brief, animals were anesthetized using Zoletil 50 (tiletamine plus zolazepam; Virbac Laboratories, Carros, France) via intraperitoneal injection. A midline cervical incision was made, and both isolated common carotid arteries were tightly double-ligated with 3-0 silk sutures. Normal group animals were subjected to the same manipulation as in the BCCAO group without ligation. The mean body temperature of the rat was maintained at 37 ± 0.5 °C with a heating lamp.

### 4.5. Animal Experimental Design

Animals were divided into experimental groups (*n* = 8): (1) the normal and (2) the BCCAO group + equal volumes of vehicle; (3) BCCAO + CHRT-2 200 mg/kg; (4) BCCAO + CHRT-2 400 mg/kg; (5) BCCAO + EHRT 400 mg/kg; and (6) BCCAO + EGB 100 mg/kg. Oral gavage treatment was started 2 d after surgery and continued for 30 d. Behavioral tests were performed within 24 h of oral administration of the respective drugs.

### 4.6. Spontaneous Alternation Behavior Test

Working memory function and exploration behavior were evaluated using the spontaneous alternation test in a single session of Y-maze [50]. Three equal arms of the Y-shape (25 × 15 × 50 per arm) were placed at equal angles with a central area. Each animal was placed at the edge of one maze and allowed to explore freely for 10 min after a habituation phase of 30 s. In each test, the spontaneous alternations were recorded visually by a person blinded to the experiment. An arm entry was scored when a rat placed four paws within that arm. Spontaneous alternation was determined when entry into the three arms occurred on consecutive choices in triplet sets (e.g., C–A–B, B–C–A, and A–B–C). The spontaneous alternation behavior was calculated using the following equation:

% Alternation = ((number of alternations)/(total number of arm entries − 2)) × 100.(2)

### 4.7. Novel Object Recognition Test

This test is used to assess the neophiliac tendency of rats to novel objects than familiar objects, and it was performed according to a previously described protocol [51]. In the acclimatization phase, each animal was allowed to explore the open field arena (80 × 80 × 25 cm (height) black box) for 5 min without objects. In the first trial, two identical objects were placed in two opposite corners of the testing arena, and the animal was allowed to explore the two objects for 10 min, during which the amount of time explored by the animal was scored. After 24 h, in the second trial, the animal was placed into the arena again, and one of the identical objects (familiar (F)) supplied in the first trial was replaced with a new identical object (new (N)). The amount of time spent exploring each object at a distance of less than 2 cm was recorded for 10 min. Climbing over or sitting on each object was excluded. The analysis of object exploration time and the discrimination ratio was performed using the formula, total N time/(N time + F time) × 100, for each experimental group. The two identical objects and the arena were cleaned with 70% ethanol for each trial.

### 4.8. Measurement of ACh Level and AChE Activity Assay

The ACh level and AChE activity in the homogenized hippocampal tissue were determined using an ACh and AChE activity assay kit (US Biomax Inc, Denwood, MD, USA) according to the manufacturer’s assay procedure. The yellow color formed by action of AChE was quantified at 570 nm using a UV spectrophotometer (Benchmark Plus; Bio-Rad Laboratories, Hercules, CA, USA).

### 4.9. Nissl Staining and Immunohistochemistry

At the end of the experimental period, all rats were sacrificed under anesthesia, and brain tissues of eight rats were immediately isolated and stored at −80 °C for further analysis. Whole brains of three rats from each group were fixed by immersion in 4% paraformaldehyde for 24 h. Trimmed brain tissue was rinsed under running tap water for 7 h and embedded in paraffin after graded ethanol dehydration; then, 4-μm-thick paraffin sections were prepared. The slides were deparaffinized with xylene and hydrated through a descending ethanol series (dipped in 100%, 80%, 70%, or 50% ethanol), and then washed with distilled water (DW). For Nissl staining, the slides were dipped in 0.5% cresyl violet (Nissl) stain solution for 1 min and washed with DW. Sections were dehydrated through graded ethanol and then mounted using mounting medium (Thermo Fisher Scientific, Waltham, MA, USA) for permanent preservation. For the immunohistochemical analysis, aspecific labeling was blocked with Block solution (CAS, Invitrogen, Carlsbad, CA, USA) at 37 °C for 60 min. Sections were subsequently incubated with rabbit anti-Iba-1 antibody (1:200, Cat. No. 019-19741, Wako Pure Chemical Ind, Osaka, Japan) at 4 °C overnight, and then incubated with a horseradish peroxidase (HRP)-conjugated Envision dual peroxidase kit (Dako, Glostrup, Denmark) for 30 min at room temperature (RT) as a secondary antibody, before adding peroxidase substrate 3,3′-diaminobenzidine (DAB) solution (diaminobenzidine, Dako, Glostrup, Denmark). As a negative control, brain tissue sections were incubated with serum from non-immunized animals, instead of the primary antibody. All sections were visualized with a DP59 (Olympus, Tokyo, Japan) camera connected to a microscope (Olympus, Tokyo, Japan) at an original magnification of 400×. Images were recorded and analyzed with the Image J Java-based image processing program (NIH, Bethesda, MD, USA). The intensity of the immunhistochemical staining was analyzed by comparing each brain section per group using an image analysis system (Image J, NIH, MD, USA). Positively labeled cells were identified using the standard color threshold algorithm of the Image J software (software version 1.52, NIH, USA). The pixel intensity and area were then measured, and the data were expressed as immunoreactivity intensity per field of the selected region.

### 4.10. Sodium Dodecyl Sulfate (SDS)-PAGE Analysis

Hippocampal tissues were lysed with radioimmunoprecipitation assay (RIPA) buffer (pH 7.5) (Pierce Biotechnology, Rockford, IL, USA). Equal amounts of 30 µg of protein using a bicinchoninic acid (BCA) kit (Bio-Rad, Hercules, CA, USA) were separated in SDS polyacrylamide gels, transferred on a Trans-Blot Turbo transfer system (Bio-Rad, USA), and blocked with 5% non-fat milk in Tris-buffered saline with Tween (TBST) solution for non-specific binding. The membranes were probed using as primary antibodies rabbit anti-phospho-p38 MAPK (1:1000), anti-p38 MAPK (1:1000), anti-phospho-ERK (1:1000), anti-ERK (1:1000), anti-phospho-JNK (1:1000), anti-JNK (1:1000), and mouse anti-β-actin (1:3000) (Cell Signaling Technology, Danvers, MA, USA), and then incubated with an HRP-conjugated anti-rabbit or mouse secondary antibody for one hour at RT. Antibody-specific protein signals on polyvinylidene fluoride (PVDF) membranes were captured using an image analyzer (Las-3000, Fuji Photo, Tokyo, Japan) and the relative optical density (OD) of immunolabeled protein bands was evaluated using the Image J program (software, NIH, Bethesda, MD, USA). Band optical density (OD) was measured using the Image J software and normalized to β-actin or total ERK, JNK, and p38MAPK, and was expressed as the relative protein level compared with the control (arbitrary units). The approximate molecular weight of each protein was determined using internal molecular weight standards (Bio-Rad, Hercules, CA, USA). β-actin, a housekeeping protein, was used as the loading control.

### 4.11. Chemicals and Reagents for HPLC Analysis

The standard components of geniposide, coptisine, berberine, and palmatine (ChemFaces Biochemical Co., Ltd., Wuhan, China); and baicalin, baicalein, and wogonin (Sunny Biotech Co., Ltd., Shanghai, China) were purchased, and the purities of these components were ≥98.0% as determined by HPLC analysis. The analytical-grade solvents (water and acetonitrile) were obtained from J. T. Baker Chemical Co. (Phillipsburg, NJ, USA); phosphoric acid and SDS were obtained from Sigma-Aldrich (St. Louis, MO, USA).

### 4.12. Chromatographic Analysis

To perform the simultaneous determination of the seven standard components, an HPLC system (Waters Alliance e2695, Waters Corp., Milford, MA, USA) equipped with an auto sample injector, column oven, pump, and PDA detector (#2998; Waters Corp.) was used. Empower (version 3; Waters Corp) software was used to acquire and process the data. The separation of the seven components was conducted with an analytical column (Gemini C18, 250 × 4.6 mm, 5 μm) (Phenomenex, Torrance, CA, USA) maintained at 30 °C. The mobile phases consisted of 10% (*v*/*v*) acetonitrile in 0.2% SDS with 0.02% phosphoric acid (A) and acetonitrile (B). The gradient conditions were as follows: 10–40% B for 0–20 min, 40–50% B for 20–40 min, 50–100% B for 40–50 min, and 100% B for 50–57 min. The injection volume was 10 µL, and the flow rate was 1.0 mL/min.

### 4.13. Statistical Analysis

Data were expressed as means ± standard error of mean (SEM), and one-way analysis of variance (ANOVA) tests were used to detect significant differences between the normal control and the treatment group. All statistical tests were performed using GraphPad Prism 7.0 (GraphPad Software, San Diego, CA, USA). Tukey’s test was used for multiple comparisons. Differences with *p* < 0.05 were considered statistically significant.
